# Mycolactone reveals the substrate-driven complexity of Sec61-dependent transmembrane protein biogenesis

**DOI:** 10.1242/jcs.198655

**Published:** 2017-04-01

**Authors:** Michael McKenna, Rachel E. Simmonds, Stephen High

**Affiliations:** 1 Division of Molecular and Cellular Function, School of Biological Sciences, Faculty of Biology, Medicine and Health, University of Manchester, Manchester Academic Health Science Centre, Michael Smith Building, Manchester M13 9PT, UK; 2 Department of Microbial Sciences, School of Bioscience and Medicine, Faculty of Health and Medical Sciences, University of Surrey, Guildford GU2 7XH, UK

**Keywords:** Endoplasmic reticulum, Membrane protein, Mycobacterium ulcerans, Mycolactone, Protein translocation, Sec61

## Abstract

Mycolactone is the exotoxin virulence factor produced by *Mycobacterium ulcerans*, the pathogen responsible for Buruli ulcer. The skin lesions and immunosuppression that are characteristic of this disease result from the action of mycolactone, which targets the Sec61 complex and inhibits the co-translational translocation of secretory proteins into the endoplasmic reticulum. In this study, we investigate the effect of mycolactone on the Sec61-dependent biogenesis of different classes of transmembrane protein (TMP). Our data suggest that the effect of mycolactone on TMP biogenesis depends on how the nascent chain initially engages the Sec61 complex. For example, the translocation of TMP lumenal domains driven by an N-terminal cleavable signal sequence is efficiently inhibited by mycolactone. In contrast, the effect of mycolactone on protein translocation that is driven solely by a non-cleavable signal anchor/transmembrane domain depends on which flanking region is translocated. For example, while translocation of the region N-terminal to a signal anchor/transmembrane domain is refractive to mycolactone, C-terminal translocation is efficiently inhibited. Our findings highlight the diversity of Sec61-dependent translocation and provide a molecular basis for understanding the effect of mycolactone on the biogenesis of different TMPs.

## Abstract (precis)

**Highlighted Article:** The exotoxin mycolactone interferes with the biogenesis of the majority of transmembrane proteins and its actions highlight differences in how distinct classes of these proteins initially engage the Sec61 translocon.

## INTRODUCTION

The exotoxin mycolactone is produced by *Mycobacterium ulcerans* and is the causative agent of Buruli ulcer; a disease characterised by necrotic skin ulcers and immunosuppression (George et al., 1999; Silva et al., 2009; Walsh et al., 2011). Mycolactone is linked to the underproduction of several key proteins involved in the inflammatory response (Pahlevan et al., 1999; Simmonds et al., 2009; Torrado et al., 2007) and the control of blood coagulation (Ogbechi et al., 2015) as a direct result of its effect on the Sec61 complex at the endoplasmic reticulum (ER) (Baron et al., 2016; Hall et al., 2014; Ogbechi et al., 2015; McKenna et al., 2016).

Secretory proteins contain a cleavable hydrophobic N-terminal signal sequence that interacts with the signal recognition particle (SRP) upon emerging from the ribosomal exit tunnel (Blobel and Dobberstein, 1975; Walter et al., 1981). SRP binding allows the ribosome–nascent-chain complex to be delivered to the ER via an interaction with the SRP receptor (Gilmore et al., 1982a,b), and the complex is then transferred to the Sec61 complex. Some nascent secretory proteins insert into the Sec61 complex with their N-terminal signal sequence in a looped, or ‘hairpin’, conformation (Mothes et al., 1994; Voorhees and Hegde, 2016), and this insertion precedes translocation of their mature domain into the ER lumen (Görlich et al., 1992). Importantly, these sequences must be sufficiently hydrophobic to destabilise the hydrophobic interactions between transmembrane domains 2 and 7 of the core Sec61α subunit (of which there are two human isoforms, SEC61A1 and SEC61A2), and thereby open what is known as the ‘lateral gate’ (Trueman et al., 2012; Voorhees and Hegde, 2016). Mycolactone does not interfere with SRP-dependent delivery of secretory proteins to the ER but rather prevents their co-translational translocation, most likely by stabilising the Sec61 complex in a closed conformation (McKenna et al., 2016) by interacting near the lumenal plug of Sec61α (Baron et al., 2016).

A second major group of proteins that are initially targeted to the ER are the transmembrane proteins (TMPs) that, with the exception of tail-anchored proteins (Kutay et al., 1995), also depend on both SRP and the Sec61 translocon for entry into the ER (Cross et al., 2009b; High et al., 1993; Oliver et al., 1995). Following their initial delivery to the Sec61 translocon, TMP biogenesis involves, firstly, translocation of a hydrophilic region of the polypeptide into the ER lumen and, secondly, the stable integration of the polypeptide into the ER lipid bilayer. TMPs contain at least one transmembrane domain (TMD) that interacts with the Sec61 translocon, halting full translocation and mediating release of the TMP into the lipid phase via the lateral gate (Martoglio et al., 1995; Van den Berg et al., 2004). Consistent with its effect at the Sec61 translocon, mycolactone does not affect the membrane insertion of tail-anchored proteins but does affect the integration of the TMPs TNFα (also known as TNF) (Hall et al., 2014) and thrombomodulin (Ogbechi et al., 2015).

Single-pass TMPs can be sub-divided into three main classes based on whether or not they possess a cleavable N-terminal signal sequence, and their final topology in the ER membrane (see Table 1). Type I TMPs contain an N-terminal signal sequence as well as an internal TMD that anchors the polypeptide in the ER membrane with its N-terminus in the ER lumen and its C-terminus in the cytosol (‘N-lumenal–C-cytosolic’). Neither type II nor type III TMPs possess an N-terminal signal sequence and so depend on a single non-cleavable internal TMD (also known as a signal anchor) to both target them to the ER and anchor them in the membrane. While type II TMPs establish an N-cytosolic–C-lumenal topology, type III TMPs have an N-lumenal–C-cytosolic topology (Goder and Spiess, 2001).

**Table 1.**
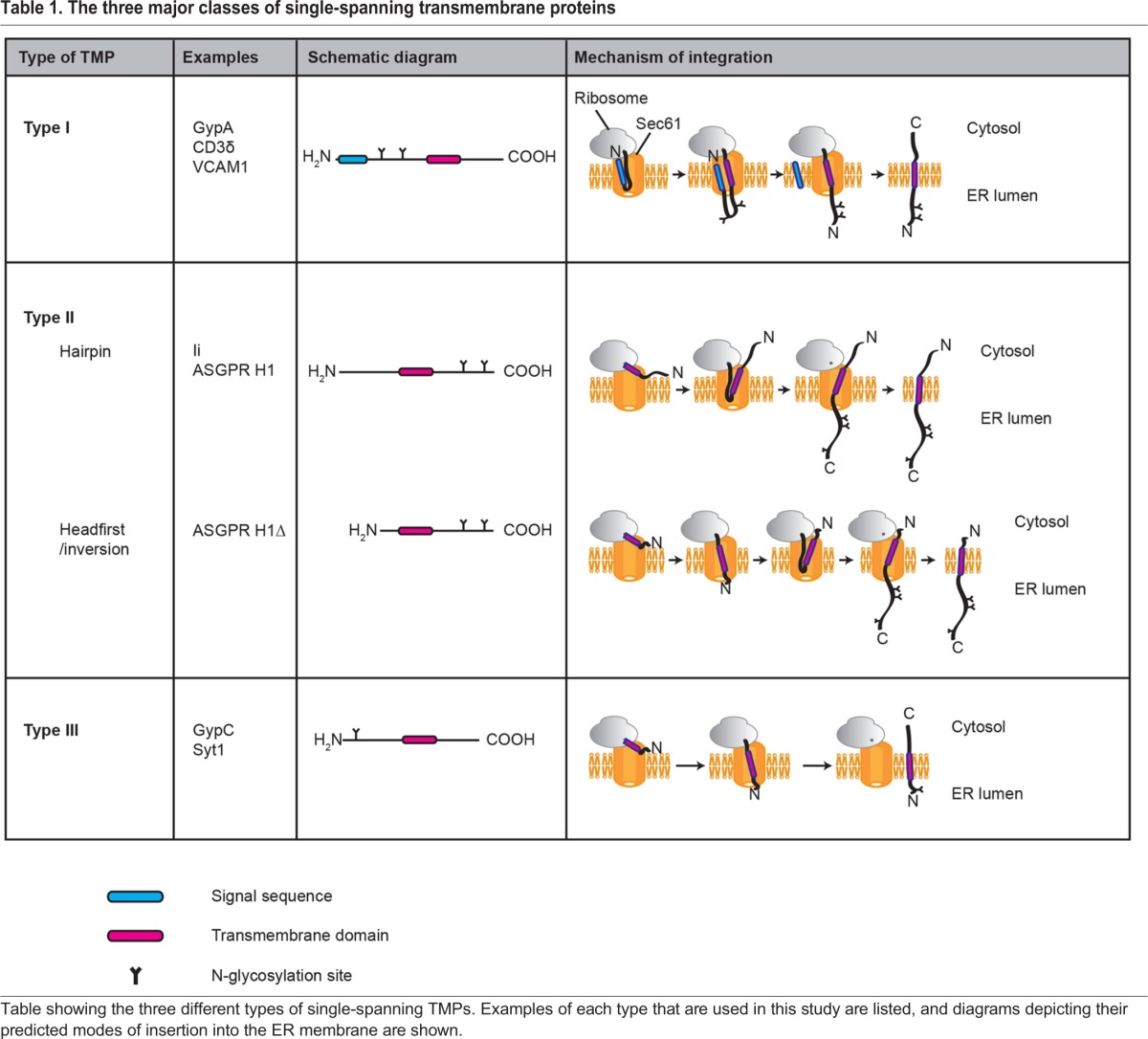
**The three major classes of single-spanning transmembrane proteins**

The final topology of these different TMPs appears to be determined by their interactions with the Sec61 translocon during their biosynthesis. For example, type III TMPs favour a ‘headfirst’ mode of insertion into the Sec61 complex, where N-terminal translocation into the ER lumen occurs as soon as the TMD emerges from the ribosomal exit tunnel (Kida et al., 2000). Type II TMPs with sufficiently short (<20 residue) N-terminal domains may also enter the Sec61 translocon headfirst, with their N-terminus initially exposed to the ER lumen, before fully inverting to adopt their correct final (N-cytosolic–C-lumenal) topology (Devaraneni et al., 2011). Type II TMPs with longer (>20 residue) N-terminal domains, such as the mycolactone-sensitive TNFα (Hall et al., 2014), are most likely to engage with the translocon as a hairpin, where the N-terminus remains exposed to the cytosol and on-going translation provides the force necessary to translocate the C-terminal domain into the ER lumen (Kocik et al., 2012). Since type I TMPs possess an N-terminal signal sequence, it is assumed that the initial stages of their biogenesis are mechanistically similar to the full translocation of secretory proteins (Walter and Lingappa, 1986). However, whilst it has been demonstrated that the N-terminal signal sequence of type I TMPs initially inserts into the Sec61 translocon as a hairpin (Shaw et al., 1988), there is surprisingly little additional biochemical evidence to support this hypothesis. An obvious question is therefore: how does mycolactone affect the integration of these distinct classes of TMP?

In this study, we characterise the integration of type I, II and III TMPs and identify clear differences in how these processes are affected by synthetic mycolactone A/B (hereafter referred to as mycolactone). Our findings build upon our current understanding of the inhibitory effects of mycolactone on protein translocation at the Sec61 translocon and highlight the mechanistic diversity in the Sec61-mediated translocation of nascent polypeptides across, and insertion into, the ER membrane. Based on our findings, we propose a model where, in the presence of mycolactone, Sec61 is altered such that the headfirst insertion of polypeptides can still occur, but both hairpin insertion into and polypeptide inversion within the translocon are restricted.

## RESULTS

### TMDs of the type I TMPs CD3δ and GypA can partially rescue their membrane integration in the presence of mycolactone

**Fig. 1.**
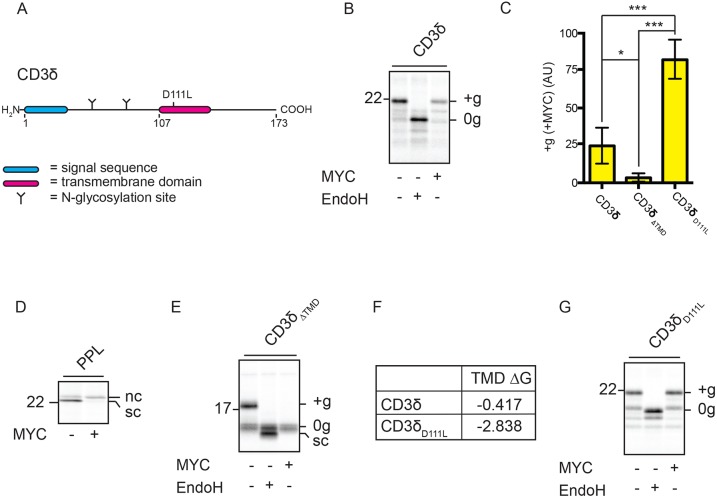
**TMDs of the type I TMPs CD3δ and GypA can partially rescue their membrane integration in the presence of mycolactone.** (A) CD3δ constructs (wild type and D111L mutant) used in this study. (B) Phosphorimage of CD3δ that had been *in vitro* translated in the absence or presence of mycolactone (MYC) and then treated with or without Endoglycosidase H (EndoH). Glycosylated (‘+g’) and non-glycosylated (‘0g’) substrate is indicated. (C) Graph showing the reduction in the amount of ‘+g’ CD3δ and related constructs in the presence of mycolactone, relative to control samples. These values were determined by dividing the quantity of ‘+g’ substrate obtained in the presence of mycolactone by the quantity of ‘+g’ substrate obtained in the absence of mycolactone and are expressed as percentages. Statistical test performed was one-way ANOVA. Error bars show mean±s.d. CD3δ, *n*=9; CD3δ_ΔTMD_, *n*=3; CD3δ_D111L_, *n*=7. ns, *P*>0.05; *P≤0.05; **P≤0.01, ***P≤0.001. (D) Translation of the secretory protein pre-prolactin (PPL) in the absence and presence of mycolactone shown for comparative purposes. Non-cleaved (‘nc’) and signal cleaved (‘sc’) substrate is indicated. (E) Translation of CD3δ_ΔTMD_ in the absence or presence of mycolactone. (F) Estimated TMD hydrophobicity values (kcal/mol) of CD3δ and CD3δ_D111L_. Hydrophobicity is based on free energy (ΔG) values, calculated using http://dgpred.cbr.su.se/ (Hessa et al., 2007). (G) Translation of CD3δ_D111L_ in the absence or presence of mycolactone.

We have previously demonstrated that co-translationally translocated secretory proteins are prevented from accessing the ER lumen due to the inhibitory effect of mycolactone at the Sec61 translocon (Hall et al., 2014; McKenna et al., 2016). Like secretory proteins, type I TMPs contain a hydrophobic cleavable signal sequence at their N-terminus (Table 1). In addition, type I TMPs contain a second hydrophobic domain (their TMD) that ultimately spans the ER membrane to generate an integral membrane protein with an N-lumenal–C-cytosolic topology (High and Dobberstein, 1992). Using a reconstituted *in vitro* system, we tested the T-cell surface glycoprotein CD3 delta chain (CD3δ, encoded by *CD3D*; Fig. 1A) for its ability to integrate into ER-derived canine rough microsomes (RMs) in the presence and absence of mycolactone. As a type I TMP, successful insertion of CD3δ is indicated by modification of its two endogenous N-glycosylation sites contained within the region N-terminal to the TMD (Fig. 1A). In the presence of mycolactone, we observed a substantial reduction in the amount of glycosylated CD3δ, but note that a small but significant proportion of glycosylated substrate persists (Fig. 1B,C; Fig. S1A). This behaviour is distinct from the complete block on translocation of secretory proteins that we had tested previously (Hall et al., 2014; McKenna et al., 2016) (Fig. 1D). Since only the membrane fractions are analysed from these integration assays, a concomitant increase in non-integrated substrate upon incubation with mycolactone is not necessarily observed. Hence, non-glycosylated species may represent either CD3δ that is peripherally membrane-associated or that is integrated but not glycosylated.

We conclude that the partial persistence (∼25%) of glycosylated CD3δ in the presence of mycolactone could be due to the inherent properties of either its N-terminal signal sequence or its TMD. To test this, we first truncated CD3δ at residue 107 to remove the TMD and thus yield an artificial secretory protein (CD3δ_ΔTMD_). Glycosylation of CD3δ_ΔTMD_ is completely inhibited by mycolactone (Fig. 1C,E), consistent with our previous observations using bona fide secretory proteins (McKenna et al., 2016) (Fig. 1D). We therefore addressed the potential role of the CD3δ TMD by introducing a point mutation to increase its net hydrophobicity (CD3δ_D111L_; Fig. 1F). We speculated that this change might enhance the ability of the TMD to overcome a mycolactone-stabilised closed conformation of Sec61 (Junne et al., 2015; Voorhees and Hegde, 2016; see also Introduction). Strikingly, we observed almost no loss in CD3δ glycosylation in the presence of mycolactone following this single amino acid substitution (Fig. 1C,G). We made similar observations regarding the mycolactone sensitivity of a second naturally occurring type I TMP, glycophorin A (GypA; Figs S1A-E). Together, these data demonstrate that type I TMPs are subject to mycolactone-dependent inhibition of the Sec61 translocon but, in contrast to the secretory proteins that we have studied previously, their subsequent TMD can influence this process.

### ER integration of the type I TMP CD3δ in the presence of mycolactone is driven by its TMD

**Fig. 2.**
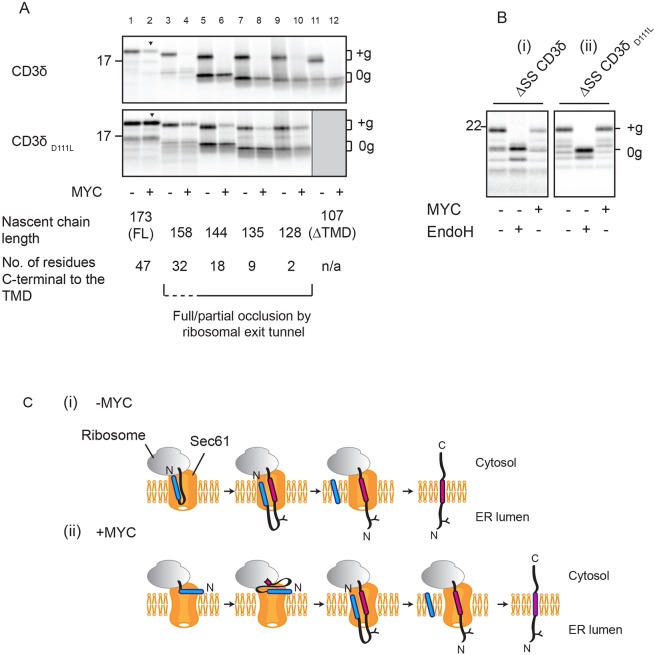
**ER integration of the type I TMP CD3δ in the presence of mycolactone is driven by its TMD.** (A) Truncated mRNAs coding for CD3δ (top panel) and CD3δ_D111L_ (bottom panel) and lacking stop codons translated in the absence or presence of mycolactone (MYC) without puromycin-mediated release. The nascent chain length of each truncation is shown, as well as the number of residues synthesised C-terminal to the TMD to provide an estimate of its distance from the peptidyl-transferase centre (PTC) of the ribosome. Truncations where all or part of the TMD is likely obscured by the ribosomal exit tunnel (based on Cabrita et al., 2016) are indicated by the bracketed area. CD3δ_158_ is encompassed by a dashed bracket, since its TMD is likely on the border of having just fully emerged from the ribosomal exit tunnel. Arrowheads indicate maximal glycosylation resulting from the TMD-dependent rescue of integration in the presence of mycolactone. (B) Versions of (i) CD3δ and (ii) CD3δ_D111L_ lacking signal sequences (ΔSS) translated in the absence and presence of mycolactone, without or without subsequent EndoglycosidaseH (EndoH) treatment. (C) Predicted mechanism of type I TMP integration in the absence (i) and presence (ii) of mycolactone. Other symbols are as defined in Fig. 1 legend. ‘+g’, glycosylated; ‘0g’, non-glycosylated; ‘C’, C-terminus; FL, full length; ‘N’, N-terminus.

On the basis of their similarity to secretory proteins, it is generally assumed that the signal sequence of type I TMPs is sufficient to both co-translationally target the nascent chain to the Sec61 translocon and to enable the subsequent translocation of its lumenal domain across the ER membrane. Here, the TMD simply acts as a ‘stop transfer’ sequence, halting further translocation of the type I TMP and enabling its release into the ER membrane (Walter and Lingappa, 1986) (Fig. 2Ci). The data presented in Fig. 1, however, suggest the TMD does not simply provide a stop-transfer sequence but indicate that in the presence of mycolactone the TMD can actively promote translocation of the type I TMP lumenal domain. To further investigate the roles of the signal sequence and TMD, we generated a series of C-terminally truncated versions of the CD3δ-encoding mRNA that lack stop codons and so produce ribosome-trapped nascent chains that reflect different stages of biogenesis (Gilmore et al., 1991). In the absence of mycolactone, each of these truncations is capable of efficient membrane integration/translocation (Fig. 2A, top panel, lanes 1, 3, 5, 7, 9, 11), even when the TMD is predicted to be fully or partially obscured by the ribosomal exit tunnel (Cabrita et al., 2016) (Fig. 2A, bracketed area), or is missing completely (Fig. 2A, lane 11). These findings support a model in which the signal sequence of this type I TMP is normally sufficient for both ER targeting and translocation (Fig. 2Ci). In the presence of mycolactone, however, N-glycosylation is only detectable when CD3δ is 173 residues long (CD3δ_173_; i.e. full length), and even then at a reduced level compared to that in the presence of a vehicle control (Fig. 2A, top panel; cf. lanes 1 and 2). This suggests that for CD3δ_173_, _­_the TMD has emerged sufficiently far from the ribosome to enable a productive interaction with the Sec61 translocon, and we speculate that this interaction can partially counteract the inhibitory effect of mycolactone. Similarly, for the equivalent truncations of CD3δ_D111L_, we see maximal rescue of translocation with the 173-residue protein (Fig. 2A, bottom panel, see arrowhead). Based on the study by Cabrita et al., 2016, we speculate that although the entire TMD of CD3δ_158_ is beyond the ribosomal exit tunnel (Fig. 2A, see dashed fraction of bracket), it has not emerged sufficiently to form a productive interaction with the Sec61 translocon and therefore remains sensitive to mycolactone (Fig. 2A, lane 4). We assume that the low levels of glycosylation of shorter truncations of CD3δ_D111L_ in the presence of mycolactone reflect the spontaneous release of some nascent chains from the ribosome (Hentzen et al., 1972).

In the case of multi-pass TMPs, TMDs of insufficient hydrophobicity can enter the translocon and remain there until a second TMD arrives, at which point the two TMDs co-operatively open the lateral gate of the translocon and exit as a pair (Meindl-Beinker et al., 2006; Pitonzo et al., 2009). To investigate if the signal sequence and TMD of CD3δ might behave in a similar way, the signal sequence was removed from CD3δ and the CD3δ_D111L_ variant, and the integration efficiency examined. Both of these polypeptides lacking the signal sequence (ΔSS) are N-glycosylated (Fig. 2Bi,Bii), demonstrating that they retain the capacity to be targeted to the ER and inserted in the correct (N-lumenal–C-cytosolic) topology. Furthermore, the extent to which the integration of each construct was inhibited by mycolactone was qualitatively similar to their respective signal-sequence-containing versions (cf. Fig 1B,G). We therefore conclude that the extent of mycolactone sensitivity of CD3δ integration is primarily determined by its TMD.

These findings lead us to propose that some type I TMPs can employ an alternative mechanism for ER translocation in the presence of mycolactone (Fig. 2Cii), thereby accounting for the portion of substrate that successfully integrates *in vitro*. In this instance, the signal sequence is sufficient to target the translating ribosome to the ER (McKenna et al., 2016) but is unable to mediate the translocation of the lumenal domain. Instead, as translation continues, our data suggest that the polypeptide accumulates on the cytosolic side of the ER until its TMD can interact productively with the Sec61 translocon to retrospectively enable translocation of the lumenal domain. While we assume that the signal sequence and TMD of such type I TMPs engage the same Sec61 translocon during this process (Gogala et al., 2014), we cannot rule out the possibility that the TMD engages a second translocon that is distinct from the one that has been unsuccessfully engaged by the signal sequence.

### The large N-terminal domain of the type I TMP VCAM1 results in a complete block of its membrane integration with mycolactone

**Fig. 3.**
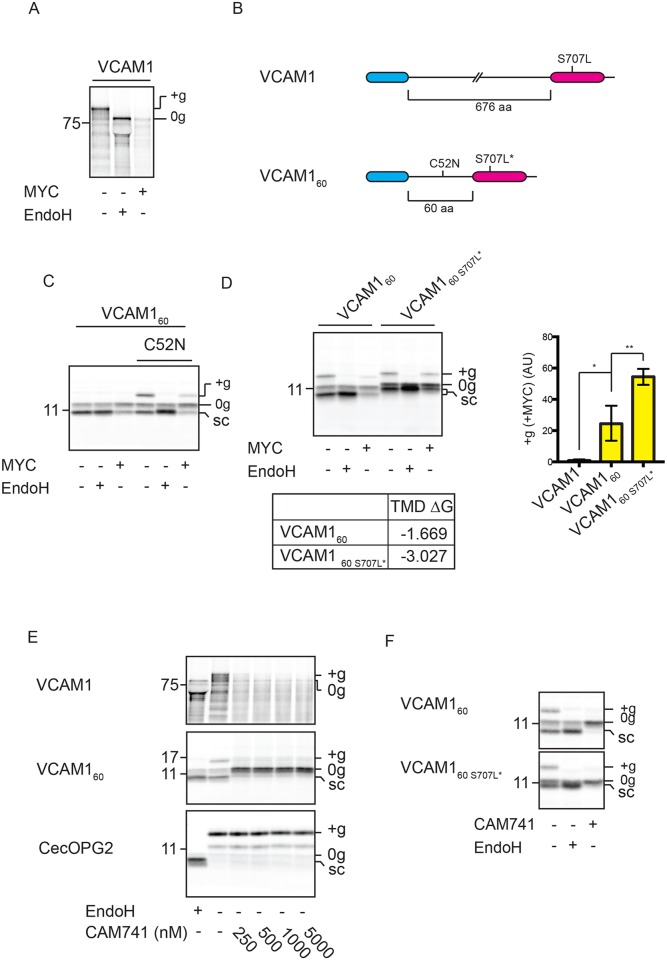
**The large N-terminal domain of the type I TMP VCAM1 results in a complete block of its membrane integration by mycolactone.** (A) VCAM1 translated in the absence or presence of mycolactone (MYC) and treated with EndoglycosidaseH (EndoH). (B) VCAM1 and VCAM1_60_ constructs (wild type and S707L/S707L* mutants) used in this study. (C) VCAM1_60_ and a version containing an artificial N-glycosylation site (C52N) translated in the absence or presence of mycolactone, without or without EndoH. (D) VCAM1_60_ and a variant with a more hydrophobic TMD (VCAM1_60 S707L*_) translated in the absence or presence of mycolactone, without or without subsequent EndoH treatment. Estimated TMD hydrophobicities (kcal/mol) are indicated in D. Graph shows the reduction in the amount of ‘+g’ VCAM1_60_ and VCAM1_60 S707L*_ in the presence of mycolactone, relative to control samples, as described in the legend to Fig. 1. is also shown in D (graph). The statistical test performed was one-way ANOVA. Error bars show mean±s.d. VCAM1, *n*=3 VCAM1_60_, *n*=4; VCAM1_60 S707L*_, *n*=3). *P*-values are as defined in Fig. 1 legend. (E) Translation of VCAM1, VCAM1_60_ and the secretory protein cecropin, possessing a C-terminal opsin tag (CecOPG2), performed with increasing concentrations of CAM741 or an equivalent volume of DMSO (‘−’). (F) VCAM1_60_ and VCAM1_60 S707L*_ translated in the absence or presence of 250 nM CAM741. Other symbols are as defined in Fig. 1 legend.

The regions separating the signal sequences and TMDs of both CD3δ and GypA are comparatively short (83 and 68 residues, respectively). For this reason, we chose to study the effect of mycolactone on the membrane integration of vascular cell adhesion protein 1 (VCAM1), which possesses an equivalent region of over 600 residues. Notably, we observed no membrane integration of VCAM1 in the presence of mycolactone (Fig. 3A; Fig. S1A), and hypothesised that its large lumenal domain may explain the lack of a mycolactone-resistant pool. To this end, we generated a shorter version of VCAM1 with only 60 residues separating its signal sequence and TMD (see Fig. S2B and Fig. 3B, VCAM1_60_). Strikingly, we now observed a partial rescue of membrane integration of VCAM1_60_ in the presence of mycolactone, as indicated by signal sequence cleavage and modification of an artificially engineered N-glycosylation site (Fig. 3C, C52N). Furthermore, this TMD-dependent effect is enhanced when the hydrophobicity of the VCAM1_60_ TMD is increased by altering a single amino acid residue (Fig. 3D). In contrast, this more hydrophobic TMD has no effect on the mycolactone-sensitivity of full-length VCAM1 (Fig. S2A). Upon extending the region between the signal sequence and TMD of VCAM1_60_ by an additional 50 residues (VCAM1_110_), the partial rescue of protein integration in the presence of mycolactone is lost, even in combination with the more hydrophobic TMD (Fig. S2B,C). These findings highlight the importance of both TMD hydrophobicity and lumenal domain size in conferring sensitivity to mycolactone.

Our characterisation of VCAM1 as a mycolactone-sensitive substrate allows us to draw comparisons with the mechanisms of CAM741, which is a well-defined small molecule that inhibits Sec61-dependent translocation in a highly substrate-specific manner (Besemer et al., 2005). Previous studies have demonstrated that VCAM1 integration is selectively and efficiently blocked by CAM741 on the basis of its signal sequence composition (Harant et al., 2006). In contrast to mycolactone, CAM741 efficiently inhibits the translocation of both VCAM1 and VCAM1_60_, but not of a third – the CAM741-resistant substrate CecOPG2, an opsin-tagged construct derived from *Hyalophora cecropia* cecropin-A (Johnson et al., 2013) (Fig. 3E). Furthermore, increasing the net hydrophobicity of the VCAM1_60_ TMD does not reduce the effectiveness of CAM741 inhibition (Fig. 3F). Therefore, our studies of VCAM1 highlight key differences in the inhibitory mechanisms of the polyketide mycolactone and the cyclopeptolide CAM741 (see Discussion).

### Mycolactone does not interfere with type III TMP integration

**Fig. 4.**
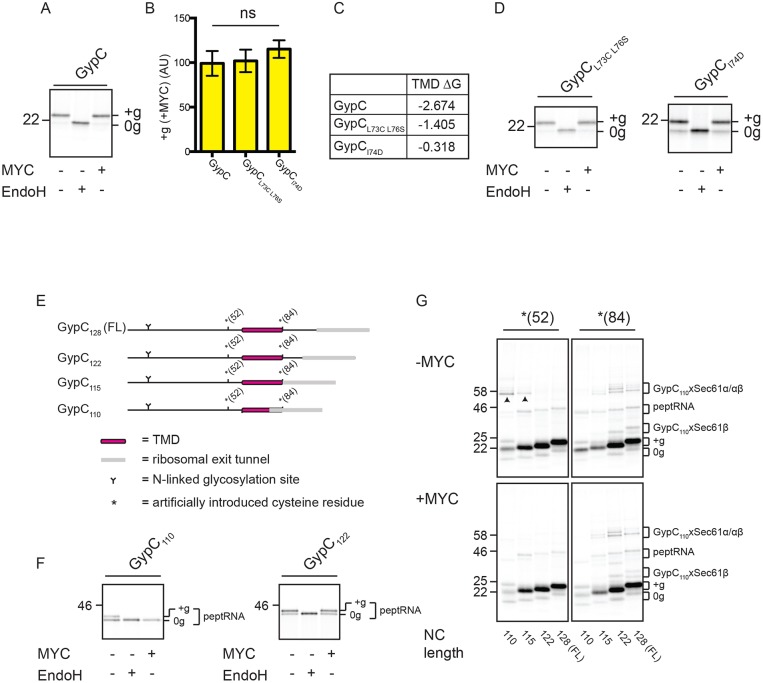
**Mycolactone does not interfere with type III TMP integration.** (A) Translation of GypC in the absence or presence of mycolactone (MYC), followed by subsequent treatment with EndoglycosidaseH (EndoH). (B) Graph shows change in the amount of glycosylated (+g) GypC and related constructs in the presence of mycolactone, relative to control samples as described in the legend to Fig. 1. The statistical test performed was one-way ANOVA. Error bars show mean±s.d. GypC, *n*=10; others, *n*=3. Ns, not significant. (C) Estimated TMD hydrophobicities (kcal/mol) of GypC and related constructs. (D) Translation of two variants of GypC with reduced TMD hydrophobicity. (E) GypC truncations lacking stop codons. For crosslinking experiments, truncations contained a single artificially introduced cysteine residue at either position 52 or 84, as denoted by an asterisk. (F) Truncated GypC chains synthesised in the absence or presence of mycolactone without puromycin-mediated release. The glycosylation of nascent chains when still attached to the ribosome (indicated by ‘peptRNA’) was observed. (G) Truncated GypC chains containing a single cysteine residue [either *(52) or *(84)] synthesised in the absence or presence of mycolactone without puromycin-mediated release to generate membrane integration intermediates. Samples were treated with the crosslinking reagent BMH, subjected to extraction with alkaline sodium carbonate, and analysed by SDS-PAGE. Adducts between the nascent chain and Sec61β (xSec61β) or the nascent chain and Sec61α/Sec61α and Sec61β (xSec61α/αβ) are indicated (see also Fig. S3B). Mycolactone-sensitive adducts are indicated by arrowheads. Other symbols are as defined in Fig. 1 legend. FL, full length.

We next sought to study the effect of mycolactone on a naturally occurring membrane protein that has the same final topology as a type I TMP but that lacks an N-terminal signal sequence. Glycophorin C (GypC) is a naturally occurring type III single-pass transmembrane protein (type III TMP; High and Tanner, 1987) and so depends on an internal TMD to both target the nascent polypeptide to the ER and anchor it in an N-lumenal–C-cytosolic topology within the ER membrane (see Table 1). Strikingly, the integration of GypC into RMs is unaffected by mycolactone (Fig. 4A,B; Fig. S1A). Furthermore, versions of GypC possessing point mutations that reduce net TMD hydrophobicity remain resistant to mycolactone (Fig. 4B,C,D), suggesting that hydrophobicity per se is not sufficient to explain this observation. Similar findings were also made using a second naturally occurring type III TMP, synaptotagmin 1 (Syt1) (Kida et al., 2000; Perin et al., 1991) (Fig. S3A).

To establish if mycolactone affects GypC at a stage before it has completed membrane integration, we again used truncated mRNAs to generate ribosome-trapped nascent chains (Fig. 4E). Furthermore, in order to specifically focus on the translocation status of nascent ribosome-associated GypC polypeptides, we analysed the glycosylation status of GypC peptidyl-tRNA (peptRNA) species (Borel and Simon, 1996) (Fig. 4F). Glycosylation, and hence N-terminal translocation, was observed for GypC_110_, for which the TMD is predicted to be partially obscured by the ribosomal exit tunnel (Cabrita et al., 2016), as well as GypC_122_ peptRNA, for which the TMD is likely to be fully exposed (Fig. 4E,F). In the presence of mycolactone, however, glycosylation of GypC_110_ peptRNA is lost whereas that of GypC_122_ peptRNA is maintained (Fig. 4F). GypC is therefore sensitive to mycolactone at some point during its biogenesis but achieves mycolactone-resistance when the TMD is sufficiently beyond the ribosomal exit tunnel.

To investigate the effect of mycolactone on the interacting partners of these GypC integration intermediates, we introduced a single cysteine residue at either the N-terminal [*(52)] or C-terminal [*(84)] side of its TMD (Fig. 4E). This allowed us to generate cysteine–cysteine crosslinks upon addition of bis-maleimidohexane (BMH). In the absence of mycolactone, we observed crosslinking from the N-terminal cysteine probe to Sec61α with the shortest GypC truncation constructs (GypC_110_ and GypC_115_) (Fig. 4G, top left panel, see arrowheads; Fig. S3B). These adducts are N-glycosylated (Fig. S3C) and therefore represent N-terminally translocated nascent GypC polypeptides. Furthermore, these adducts are lost upon disruption of the ribosome–nascent-chain complex following treatment with puromycin (Fig. S3D), thus demonstrating that they reflect the environment of bona fide trapped integration intermediates. Extension of the nascent chain by just seven residues (to GypC_122_) results in a concomitant loss of adducts with the N-terminal cysteine probe and an increase in crosslinking from the C-terminal cysteine probe to both Sec61α and Sec61β (Fig. 4G, top two panels; Fig. S3B). When the same analysis is performed in the presence of mycolactone, the Sec61α adducts with GypC_110_ and GypC_115_ are lost, while those of the two longer intermediates are maintained (Fig. 4G, bottom panels; Fig. S3D), consistent with the acquisition of mycolactone resistance at these longer chain lengths (Fig. 4F).

Taken together, these data show that integration of type III TMPs at the ER is highly resistant to mycolactone, even when these substrates possess TMDs of relatively modest hydrophobicity. Additionally, our data suggest that only a portion of the GypC TMD needs to be exposed in order for N-terminal translocation to occur but that these truncated polypeptides are transiently sensitive to mycolactone. Acquisition of mycolactone resistance occurs when the C-terminus of GypC is extended by just a few residues, at which point the TMD is most likely to be fully exposed and not protected by the ribosome.

### Mycolactone efficiently blocks integration of type II TMPs

While N-terminal signal sequence-driven translocation of polypeptides through the Sec61 translocon is efficiently blocked by mycolactone, its effects on the TMD-driven translocation of the substrates we have so far investigated appears to be more variable. One possible explanation for this observation is that TMDs tend to be longer and more hydrophobic than N-terminal signal sequences (von Heijne, 1985, 1986), and this may enable them to overcome the effects of mycolactone more successfully. Alternatively, the differences we observed may be determined by whether the region that is being translocated is N- or C-terminal to the hydrophobic ER-targeting sequence that engages the Sec61 complex. For example, while an N-terminal signal sequence translocates the region to its C-terminus, the TMDs of type III TMPs (Kida et al., 2000), and type I TMPs when analysed in the presence of mycolactone (this study), translocate regions that are to their N-terminus.

**Fig. 5.**
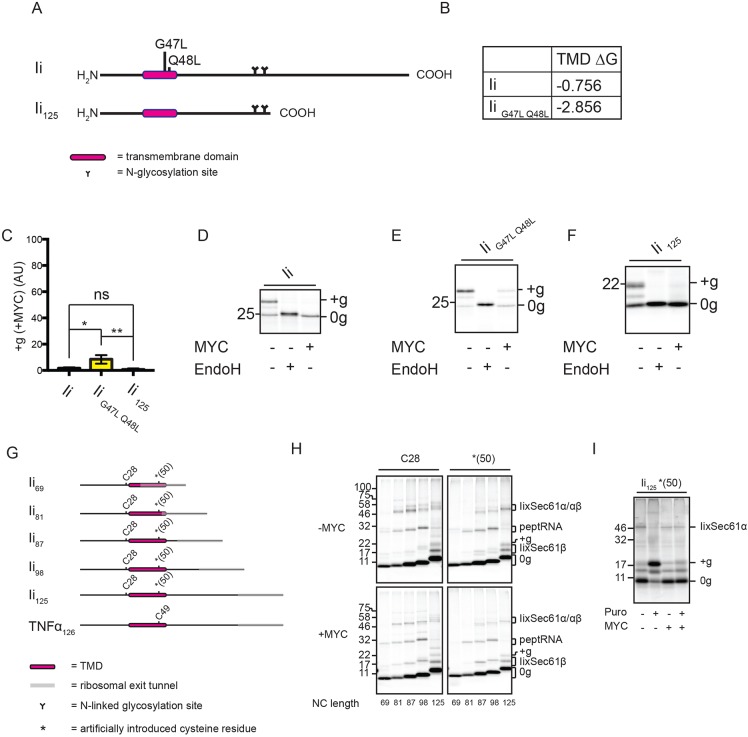
**Mycolactone efficiently blocks type II TMP integration.** (A) Full-length Ii (wild type and G47L Q48L mutant) and the Ii_125_ truncation used in this study. (B) Estimated TMD hydrophobicities (kcal/mol) of Ii and Ii_G47L Q48L_. (C) Graph shows the reduction in the amount of glycosylated (+g) Ii and related constructs in the presence of mycolactone (MYC), relative to control samples as described in the legend to Fig. 1. The statistical test performed was one-way ANOVA. Error bars show mean±s.d. (*n*=3). *P*-values are as defined in Fig. 1 legend. Translation in the absence or presence of mycolactone performed using Ii (D), Ii_G47L Q48L_ (E) and Ii_125_ (F), which was followed by treatment with EndoglycosidaseH (EndoH). (G) Ii truncations used in this study. For crosslinking experiments, truncations contained either a native cysteine residue (C28) or one that was artificially introduced [*(50)]. A truncated version of TNFα used for crosslinking analysis (as described in MacKinnon et al., 2014) is shown for comparative purposes. Crosslinking was performed on Ii truncations (H) and Ii_125_*(50) (I) and the resulting adducts are labelled as described in the Fig. 4G legend. Other symbols are as defined in Fig. 1 legend. Puro, puromycin.

We therefore studied the MHC class II-associated invariant chain (Ii; Fig. 5A), a type II TMP with a single TMD that enables the translocation of its C-terminus, resulting in a final N-cytosolic–C-lumenal topology. Despite having a long and relatively hydrophobic TMD (compared to CD3δ and GypC; cf. Figs 1F, 4C and 5B), correctly integrated Ii is barely detectable in the presence of mycolactone (Figs 5C,D; Fig. S1A). Furthermore, integration of Ii remains highly sensitive to mycolactone when the hydrophobicity of the TMD is increased to a ΔG value similar to that for GypC (cf. Figs 4C and 5B, and Fig. 5E). Likewise, mycolactone efficiently blocks the integration of Ii with a truncated C-terminus (IC_125_, Fig. 5F), thus ruling out the possibility that its sensitivity is due to the larger size of its lumenal domain.

Despite the strong inhibitory effect of mycolactone on Ii integration, we observed surprisingly little change in the crosslinking profile of ribosome-trapped nascent chains of various lengths when mycolactone is present (Fig. 5G,H). What we do find is that the full membrane integration of Ii following puromycin-mediated release from the ribosome is prevented by mycolactone. Instead, the nascent chain is retained in proximity to the translocon, as indicated by continued cross-linking to Sec61α in the presence of mycolactone (Fig. 5I). We therefore conclude that mycolactone prevents the type II TMP Ii from assuming its correct N-cytosolic–C-lumenal topology, and instead causes the nascent chain to be retained at a pre-integration step (see Discussion).

### Mycolactone sensitivity is dependent upon which TMD-flanking region is translocated

**Fig. 6.**
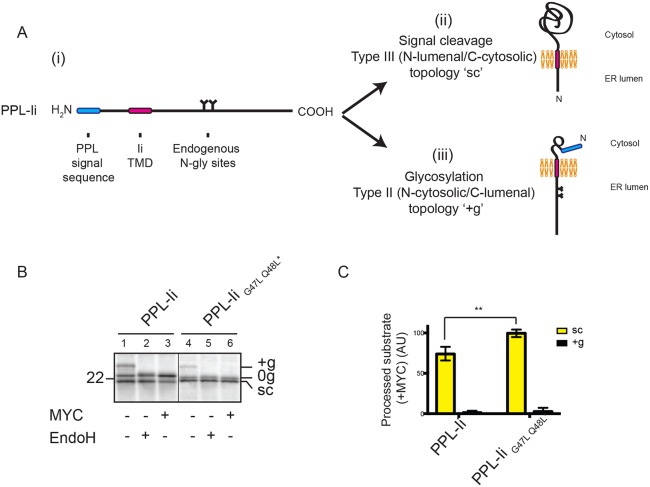
**Mycolactone sensitivity is dependent upon which TMD-flanking region is translocated.** (A) A chimeric protein containing Ii downstream of a pre-prolactin (PPL) signal sequence (i) and the two topologies it might assume following integration into RMs, depending on whether the region that is translocated is N-terminal (ii) or C-terminal (iii) of the TMD. (B) Translation of PPL-Ii and PPL-Ii_G47L Q48L*_ in the absence or presence of mycolactone (MYC), followed by treatment with EndoglycosidaseH (EndoH). Samples were analysed following immunoprecipitation of Ii. (C) Graph showing the amount of signal-cleaved (‘sc’) or glycosylated (‘+g’) substrate in the presence of mycolactone relative to control samples. These values were determined by dividing the quantity of ‘sc’ or ‘+g’ substrate obtained in the presence of mycolactone by the quantity of ‘sc’ or ‘+g’ substrate obtained in the absence of mycolactone and are expressed as percentages. The statistical test performed was two-way ANOVA. Error bars show mean±s.d. (*n*=3). *P*-values and other symbols are as defined in Fig. 1 legend.

The observations in this study with type II and type III TMPs suggest that mycolactone sensitivity may be dependent on whether the region that is translocated through the Sec61 complex lies N- or C-terminal to the TMD. These comparisons have so far relied on substrates that vary in several factors known to contribute to final protein topology, including TMD hydrophobicity, relative position of the TMD, and the number and location of charged residues flanking the TMD. To address these issues, we generated a chimeric protein that contains Ii downstream of a pre-prolactin signal sequence (PPL-Ii; Fig. 6Ai) with the intention of promoting the unnatural N-terminal translocation of this artificial protein across the ER membrane. We therefore expected to observe either cleavage of the N-terminal signal sequence (Fig. 6Aii) or N-glycosylation of the C-terminal region (Fig. 6Aiii), depending on whether the region that is translocated is N- or C-terminal to the TMD. By introducing an artificial N-glycosylation site between the signal sequence and TMD, we exclude a third possible scenario in which the chimeric protein translocates the domain N-terminal to the TMD but signal sequence cleavage does not occur (Fig. S4A,B). In the absence of mycolactone, we observed both signal-cleaved and N-glycosylated PPL-Ii (Fig. 6B, lane 1), indicating that this artificial protein assumes a mixed transmembrane topology. In the presence of mycolactone, however, almost no glycosylation is detected, yet signal sequence cleavage is still observed (Fig. 6B, lane 3, and 6C). Increasing the hydrophobicity of the TMD of PPL-Ii (see Fig. 5B) results in the complete insensitivity of signal sequence cleavage to mycolactone, whereas N-glycosylation remains almost completely blocked (Fig. 6B, lane 6, and 6C). Since the PPL signal sequence is incapable of overcoming the inhibitory effect of mycolactone (McKenna et al., 2016) (Fig. 1D), and we observed no contribution of the signal sequence to the mycolactone resistance of TMD-containing proteins (Fig. 2B), both final topologies of PPL-Ii in the presence of mycolactone must result from membrane integration enabled by the TMD. We therefore conclude that even when membrane integration in two different topologies can be driven by the same TMD, translocation of its C-terminal region is preferentially inhibited by mycolactone.

### Mycolactone traps headfirst-inserting type II TMPs in an N-lumenal–C-cytosolic topology

**Fig. 7.**
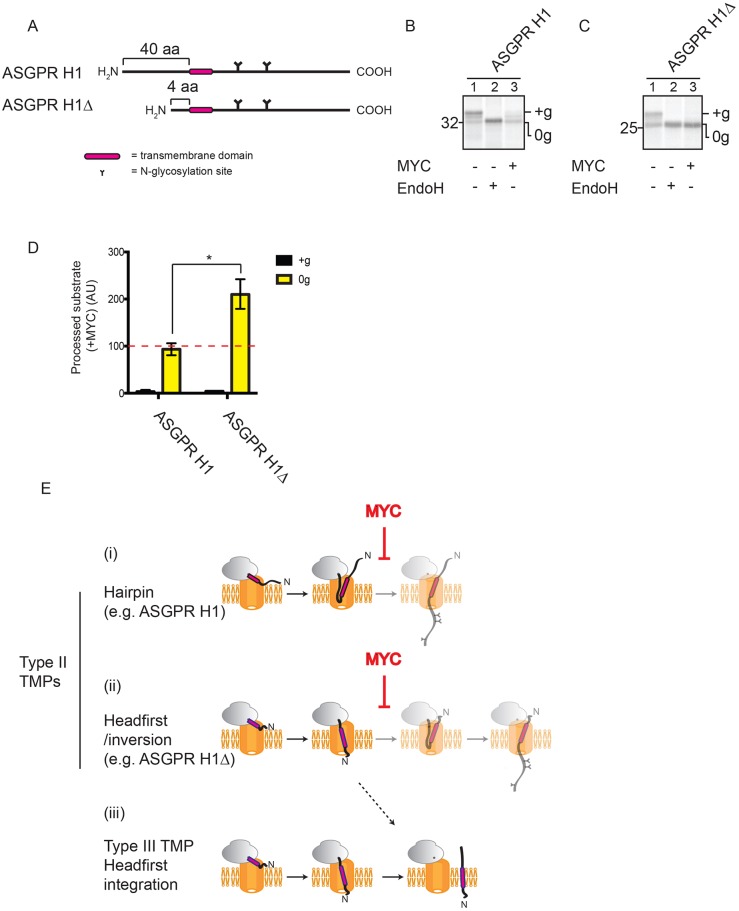
**Mycolactone traps headfirst-inserting type II TMPs in an N-lumenal–C-cytosolic topology.** (A) ASGPR H1 and ASGPR H1Δ. Translation of ASGPR H1 (B) and ASGPR H1Δ (C) performed in the absence or presence of mycolactone (MYC), followed by treatment with EndoglycosidaseH (EndoH). Membrane fractions were subjected to extraction with alkaline sodium carbonate prior to analysis. (D) Graph shows the amount of glycosylated (‘+g’) and non-glycosylated (‘0g’) ASGPR H1 and ASGPR H1Δ in the presence of mycolactone, relative to control samples. These values were determined by dividing the quantity of ‘+g’ or ‘0g’ substrate obtained in the presence of mycolactone by the quantity of '+g' or '0g' substrate obtained in the absence of mycolactone and are expressed as percentages. Dashed red line represents the value for comparative material for samples treated with a vehicle control. The statistical test performed was two-way ANOVA. Error bars show mean±s.d. (*n*=3). *P*-values are as defined in Fig. 1 legend. (E) Diagram showing type II TMPs that insert using a hairpin mechanism (i) or a headfirst/inversion mechanism (ii), as well as the headfirst insertion of type III TMPs (iii). Faded steps represent those that are prevented by mycolactone. Dashed arrow shows the predicted route taken by headfirst-inserting type II TMPs when inversion is prevented by mycolactone.

Previous studies suggest that type II TMPs can engage the Sec61 translocon in one of two ways, depending largely on the size of their N-terminal domain. Those with an N-terminal domain longer than ∼20 residues, like our model type II TMP, Ii, are generally believed to insert as a hairpin, while those with an N-terminal domain shorter than ∼20 residues are proposed to insert headfirst, like a type III TMP, before fully inverting within the translocon (Devaraneni et al., 2011; Kocik et al., 2012; Table 1). To look more closely at the effect of mycolactone on these two alternative modes of insertion of type II TMPs, we used the asialoglycoprotein receptor subunit H1 (Fig. 7A, ASGPR H1), which possesses a 40 residue N-terminal domain, in combination with a truncated form that possesses an N-terminal region of just four amino acids (Fig. 7A) (ASGPR H1Δ; Wahlberg and Spiess, 1997). Previous studies suggest that ASGPR H1 employs a hairpin mechanism for membrane insertion whilst the truncation of its N-terminal domain promotes the insertion of ASGPR H1Δ via a headfirst mechanism (Kocik et al., 2012; Wahlberg and Spiess, 1997). N-glycosylation of both ASGPR H1 and ASGPR H1Δ is strongly inhibited by mycolactone (Fig. 7B,C,D, see ‘+g’; Fig. S1A), supporting our conclusion that mycolactone efficiently prevents type II TMPs from achieving their correct N-cytosolic–C-lumenal topology. Notably, the TMD of the type II TMP ASGPR H1 is more hydrophobic than that of Ii (ΔG of −2.828 kcal/mol versus −0.756 kcal/mol), reinforcing that hydrophobicity alone is not sufficient to explain the effects we see of mycolactone on TMP biogenesis. Interestingly, the amount of non-glycosylated ASGPR H1Δ increases in the presence of mycolactone, even after the membranes are treated with alkaline sodium carbonate to remove non-integrated polypeptides (Fig. 7C, cf. lanes 1 and 3, and 7D, see ‘0g’). We therefore conclude that the non-glycosylated ASGPR H1Δ species most likely represents successfully integrated substrate that has failed to invert and hence remains in a N-lumenal–C-cytosolic topology [cf. Fig. 7Eii,Eiii; see also Wahlberg and Spiess, 1997). In contrast, the amount of non-glycosylated full-length ASGPR H1 does not increase in the presence of mycolactone under similar conditions (Fig. 7B, cf. lanes 1 and 3, and 7D, see ‘0g’), suggesting that in this case, the nascent polypeptide has simply failed to integrate (Fig. 7Ei). These results therefore suggest that type II TMPs that employ a headfirst-inversion mode of insertion into the ER may be prevented from inverting within the Sec61 translocon by mycolactone and, instead, become integrated into the ER membrane in an N-lumenal–C-cytosolic topology (Fig. 7Eii,Eiii).

## DISCUSSION

In this study, we explore the inhibitory mechanism of mycolactone at the Sec61 translocon by investigating the integration of three distinct classes of single-pass transmembrane proteins (TMPs). We conclude that mycolactone binding restricts the Sec61 complex, leading to disruption of TMP biogenesis. However, distinct classes of TMP respond differently to mycolactone, most likely reflecting the precise nature of their initial engagement at the translocon. Thus, whilst the headfirst insertion of polypeptides is unaffected, their hairpin insertion into and inversion within the translocon are inhibited.

### What makes a protein mycolactone-sensitive?

Secretory and membrane proteins are key therapeutic targets, and it is important to understand the molecular basis for the selectivity of inhibitors that affect their biogenesis (Kalies and Römisch, 2015). For the majority of small molecules known to modulate ER translocation, they appear to do so by acting directly at the Sec61 complex (Kalies and Römisch, 2015). Compounds such as eeyarestatin and apratoxin inhibit the Sec61-dependent translocation of a broad range of substrates (Cross et al., 2009a; Paatero et al., 2016). In contrast, and despite their common Sec61 target, HUN-7293-derived compounds such as CAM741 and the cotransin family display clear substrate specificity (Besemer et al., 2005; Garrison et al., 2005; Maifeld et al., 2011). Exactly why certain compounds have different substrate-specific effects on ER translocation is unclear, and it is in this context that we are studying the effects of mycolactone, which we have previously showed to have a broad ranging effect on the co-translational translocation of secretory proteins across the ER membrane (Hall et al., 2014; McKenna et al., 2016).

We now present several lines of evidence that support a model in which the inhibition of protein translocation by mycolactone is strongly influenced by how the nascent polypeptide initially engages the Sec61 translocon. Firstly, we have already shown that secretory proteins are efficiently blocked by mycolactone (Hall et al., 2014; McKenna et al., 2016). We now establish that removal of the TMDs of type I TMPs that otherwise retain some degree of resistance to mycolactone generates artificial secretory proteins that are strongly inhibited by mycolactone as a consequence of their now wholly N-terminal signal sequence-dependent ER translocation. Secondly, we show that type I TMPs can integrate into the ER via one of two mechanisms: in the absence of mycolactone, the N-terminal signal sequence is sufficient to mediate translocation of the region C-terminal to it, consistent with the generally assumed mechanism of integration for type I TMPs (Walter and Lingappa, 1986); whilst in the presence of mycolactone, translocation driven by the N-terminal signal sequence is efficiently blocked and, instead, translocation of sufficiently small lumenal domains can be mediated by the TMD. Here, we see that removal of the N-terminal signal sequence from the type I TMP CD3δ has no effect on the extent to which its membrane insertion is inhibited by mycolactone. Thirdly, although both type II and type III TMPs contain a single TMD, the former translocate their C-terminal region and are strongly inhibited by mycolactone, while the latter translocate their N-terminal region and are unaffected by mycolactone treatment. Fourthly, by generating a chimeric protein whose TMD can translocate either its N- or C-terminus, we show that C-terminal translocation is inhibited by mycolactone to a much greater extent. Lastly, we use a truncated version of ASGPR H1 as a model to study the so-called headfirst-inversion mechanism of some type II TMPs (Devaraneni et al., 2011; Kocik et al., 2012; Wahlberg and Spiess, 1997). Our data suggest that mycolactone does not prevent the headfirst insertion of this model substrate into the Sec61 complex but inhibits its subsequent inversion, most likely fixing it in a ‘type III-like’ (N-lumenal–C-cytosolic) topology. Interestingly, the N-terminal signal sequences of secretory proteins and type I TMPs have been shown to engage the Sec61 translocon in a hairpin conformation (Mothes et al., 1994; Shaw et al., 1988; Voorhees and Hegde, 2016). Taken together, our findings therefore suggest that polypeptides that initially engage the Sec61 translocon as a hairpin are effectively prevented from correctly integrating/translocating into the ER. In contrast, headfirst insertion of polypeptides into the Sec61 translocon can still occur, but those substrates that require a subsequent inversion step within the translocon are prevented from doing so.

Our conclusion that the type II TMP Ii is trapped at a ‘pre-integration’ stage by mycolactone is supported by a previous study of membrane protein biogenesis using the cotransin CT8 (MacKinnon et al., 2014). Ii closely resembles TNFα, the model substrate used in that previous study (Fig. 5G). In both cases, similarly positioned cysteine residues form adducts with components of the Sec61 translocon that are lost following full integration into the ER membrane but that are maintained in the presence of mycolactone or CT8. Moreover, mutagenesis of Sec61α and competitive binding assays have shown that CT8 and mycolactone most likely have overlapping binding sites (cf. Baron et al., 2016 and MacKinnon et al., 2014). Whilst these similarities suggest the inhibitory mechanisms of these two small molecules are related, there is notably little effect of CT8 on the biogenesis of other mycolactone-sensitive substrates, such as pre-prolactin (Maifeld et al., 2011; McKenna et al., 2016), highlighting clear differences between them. Furthermore, our investigation of the type I TMP VCAM1 distinguishes the mechanism of inhibition of mycolactone from that of the cotransin-like compound CAM741. While CAM741 blocks integration in a signal sequence-specific manner that prevents productive interactions of a subsequent TMD with the Sec61 translocon, mycolactone indiscriminately interferes with signal sequence-mediated protein translocation, but its effect can be partially overcome by sufficiently hydrophobic TMDs.

To date, the focus for identifying mycolactone-sensitive substrates in a cellular context has been on mediators of the immune response and blood anticoagulation. Both secreted mediators, such as cytokines and chemokines, as well as TMPs, such as receptors, are under-produced in cells treated with mycolactone, while cytoplasmic substrates appear to be consistently unaffected (Coutanceau et al., 2007; Hall and Simmonds, 2014; Simmonds et al., 2009). Our current study suggests that type I TMPs with N-terminal domains of more than ∼100 residues are highly sensitive to mycolactone treatment *in vitro*. Notably, most of the type I TMPs that are mycolactone-sensitive at a cellular level have N-terminal domains of a similar or larger size (cf. Hall and Simmonds, 2014), including thrombomodulin, consistent with the previously suggested mycolactone-dependent inhibition of its ER translocation (Ogbechi et al., 2015). Our limited *in vitro* analysis of type II TMPs suggests that their membrane integration is highly sensitive to mycolactone, and this is consistent with previous observations using TNFα (Hall et al., 2014). In contrast to our observations with type I and type II TMPs, we find that type III TMP integration appears to be completely resistant to mycolactone. Strikingly, the only type III TMP to be studied at a cellular level, linker for activation of T-cells family member 1 (LAT), has been previously shown to also be unaffected by mycolactone treatment (Boulkroun et al., 2010). Estimates suggest that human type III TMPs represent only ∼2% of all single-spanning membrane proteins (Uniprot), and hence the majority of single-spanning TMPs are likely to display some degree of sensitivity to mycolactone. In line with this, a recent study has reported that the majority of Sec61-dependent substrates that could be identified are sensitive to mycolactone, with single-pass TMPs being particularly enriched (Baron et al., 2016). In short, our findings provide a potential molecular basis for the complex changes in membrane protein expression that underlie the pathogenesis of Buruli ulcer.

### What can mycolactone tell us about Sec61-dependent translocation?

The substrate-specific inhibition of Sec61-dependent translocation by mycolactone that we describe in this study supports the idea that the TMD of a nascent polypeptide plays an important role in opening the Sec61 lateral gate. A recent study using native membranes concludes that full opening of the Sec61 translocon appears to occur upon the docking of even non-translating ribosomes (Pfeffer et al., 2015). In contrast, our findings align more closely with a model in which ribosome docking primes the Sec61 translocon by partially opening its lateral gate. In this model, full opening occurs only when a sufficiently hydrophobic signal sequence/TMD of the nascent polypeptide chain positionally replaces one of the Sec61α TMDs located at the lateral gate, thereby exposing the hydrophobic region to the lipid phase of the ER membrane (Voorhees and Hegde, 2016). An interesting implication of our study is that opening of the lateral gate may occur more readily when a substrate engages the translocon in a particular orientation (namely headfirst), regardless of its hydrophobicity. Recent structural studies of the engaged Sec61 translocon have greatly advanced our knowledge of ER translocation (Gogala et al., 2014; Voorhees and Hegde, 2016) but have so far only investigated a narrow range of Sec61-dependent substrates. Our findings demonstrate the substrate-driven complexity of Sec61-dependent protein translocation and therefore highlight the importance of considering a broad range of substrates in future structural studies aiming to characterise this process.

## MATERIALS AND METHODS

Synthetic mycolactone A/B was a gift from Yoshito Kishi, Harvard University, Cambridge, MA (Song et al., 2002). CAM741 was generously provided by Boehringer. Unless otherwise stated, all standard laboratory reagents were obtained from Merck or Sigma.

### DNA constructs

Unless otherwise stated, all cDNAs matched human sequences. GypC, GypA and Ii (*Mus* *musculus*; Oliver et al., 1995), PPL (*Bos taurus*; McKenna et al., 2016) and CecOPG2 (*Hyalophora cecropia*; Johnson et al., 2012) have all been described previously. VCAM1 was a gift from Hanna Harant (Ingenetix), CD3δ was a gift from Cornelia Wilson (Canterbury Christ Church University, Kent, UK), ASGPR H1 was a gift from Martin Spiess (University of Basel, Basel, Switzerland) and Syt1 (*Rattus norvegicus*) a gift from Alan Morgan (University of Liverpool, Liverpool, UK). The PPL-Ii chimera was generated by cloning Ii downstream of PPL using an engineered EcoRI site. Point mutations were generated using primers from Eurogentec. cDNAs were generated by PCR and transcribed with T7 polymerase (Promega).

### Antibodies

Rabbit antiserum against GypC has been described previously (Elliott et al., 1997). Rabbit antiserum against canine Sec61α was from Richard Zimmermann (University of Saarland, Homburg, Germany). Rabbit antisera against Sec61β and Ii were from Bernhard Dobberstein (University of Heidelberg, Heidelberg, Germany).

### *In vitro* translation and translocation assays

*In vitro* translations used rabbit reticulocyte lysate (Promega) to synthesise radiolabelled proteins in the presence of canine rough microsomes as previously described (McKenna et al., 2016), except that reaction volumes were 20 µl and were incubated at 30°C for 15 min. For crosslinking assays, translation reactions were carried out at 30°C for 10 min. All samples were then treated with 0.5 mM puromycin and incubated at 30°C for 5 min unless stated otherwise in the figure legends. For CAM741 inhibition assays, CAM741 (in DMSO) was added to give the appropriate concentration at the beginning of the reaction. The control used an equivalent volume of DMSO.

### Membrane recovery and visualisation

Membranes were recovered as described previously (McKenna et al., 2016). Unless stated otherwise in the figure legends, the membrane pellet was immediately resuspended in 30 µl SDS sample buffer [100 mM Tris-HCl, pH 6.8, 100 mM DTT, 4% (w/v) SDS, 20% (w/v) glycerol, 1% (v/v) L-methionine, 10 mM EDTA, Bromophenol Blue]. Where indicated, samples were also treated with EndoglycosidaseH (EndoH; New England Biolabs) as described by the supplier. The resulting samples were analysed and processed as described previously (McKenna et al., 2016). Data were quantified using AIDA software (Raytek), and statistical analyses (one-way ANOVA or two-way ANOVA) were performed using GraphPad (Prism). The exact sample size (*n*) for each experimental group is provided in the appropriate figure legends. In each case, *n* was defined by the number of times the substrate was tested in the same experimental system and so represents technical replicates.

### Crosslinking and carbonate extraction

After recovery, the entire pellet was resuspended in 20 µl low-salt buffer [100 mM sucrose, 100 mM KOAc, 5 mM Mg(OAc)_2_, 50 mM Hepes-KOH pH 7.9, 1 mM DTT]. Crosslinking [using BMH (1 mM final)] and carbonate extraction were performed as described previously (McKenna et al., 2016). Samples were then either analysed directly by SDS-PAGE or were first immunoprecipitated under denaturing conditions (see below).

### Denaturing immunoprecipitation

Following carbonate extraction and recovery of the membrane fraction, pellets were resuspended in 20 µl of 1% (w/v) SDS and incubated for 10 min at 70°C. Ten volumes of Triton immunoprecipitation (IP) buffer [10 mM Tris-HCl pH 7.5, 140 mM NaCl, 1 mM EDTA, 1% Triton X-100, 5 mM PMSF, 1 mM methionine] with the appropriate antiserum (1:200) were added. Samples were incubated for 15 h at 4°C with constant agitation. Protein-A–Sepharose beads (Genscript) were added to 10% (v/v), and samples were incubated at 4°C for a further 2 h. Protein-A–Sepharose beads were then recovered by spinning at 13,000* **g*** for 1 min and washed with Triton IP buffer before being heated at 70°C for 10 min in SDS sample buffer.

### Native immunoprecipitation of PPL-Ii

Instead of recovering RMs by ultracentrifugation, PPL-Ii translations (see Fig. 6B) were subjected to an anti-Ii immunoprecipitation. Following translation, nine volumes of Triton IP buffer with anti-Ii antiserum (1:200) were added. Samples were incubated for 15 h at 4°C with constant agitation. Protein-A-Sepharose beads (Genscript) were added to 10% (v/v), and samples were incubated at 4°C for a further 2 h. Protein-A–Sepharose beads were then recovered by spinning at 13,000* **g*** for 1 min and washed with Triton IP buffer before being heated at 70°C for 10 min in SDS sample buffer.

## References

[JCS198655C1] Baron L., Paatero A. O., Morel J.-D., Impens F., Guenin-Macé L., Saint-Auret S., Blanchard N., Dillmann R., Niang F., Pellegrini S. et al. (2016). Mycolactone subverts immunity by selectively blocking the Sec61 translocon. *J. Exp. Med.* 213, 2885-2896. 10.1084/jem.20160662 27821549 PMC5154940

[JCS198655C2] Besemer J., Harant H., Wang S., Oberhauser B., Marquardt K., Foster C. A., Schreiner E. P., de Vries J. E., Dascher-Nadel C. and Lindley I. J. D. (2005). Selective inhibition of cotranslational translocation of vascular cell adhesion molecule 1. *Nature* 436, 290-293. 10.1038/nature03670 16015337

[JCS198655C3] Blobel G. and Dobberstein B. (1975). Transfer of proteins across membranes. I. Presence of proteolytically processed and unprocessed nascent immunoglobulin light chains on membrane-bound ribosomes of murine myeloma. *J. Cell Biol.* 67, 835-851. 10.1083/jcb.67.3.835 811671 PMC2111658

[JCS198655C4] Borel A. C. and Simon S. M. (1996). Biogenesis of polytopic membrane proteins: membrane segments of P-glycoprotein sequentially translocate to span the ER membrane. *Biochemistry* 35, 10587-10594. 10.1021/bi960950q 8718846

[JCS198655C5] Boulkroun S., Guenin-Macé L., Thoulouze M.-I., Monot M., Merckx A., Langsley G., Bismuth G., Di Bartolo V. and Demangel C. (2010). Mycolactone suppresses T cell responsiveness by altering both early signaling and posttranslational events. *J. Immunol.* 184, 1436-1444. 10.4049/jimmunol.0902854 20042571

[JCS198655C6] Cabrita L. D., Cassaignau A. M. E., Launay H. M. M., Waudby C. A., Wlodarski T., Camilloni C., Karyadi M.-E., Robertson A. L., Wang X., Wentink A. S. et al. (2016). A structural ensemble of a ribosome-nascent chain complex during cotranslational protein folding. *Nat. Struct. Mol. Biol.* 23, 278-285. 10.1038/nsmb.3182 26926436 PMC5405865

[JCS198655C7] Coutanceau E., Decalf J., Martino A., Babon A., Winter N., Cole S. T., Albert M. L. and Demangel C. (2007). Selective suppression of dendritic cell functions by Mycobacterium ulcerans toxin mycolactone. *J. Exp. Med.* 204, 1395-1403. 10.1084/jem.20070234 17517970 PMC2118602

[JCS198655C8] Cross B. C. S., McKibbin C., Callan A. C., Roboti P., Piacenti M., Rabu C., Wilson C. M., Whitehead R., Flitsch S. L., Pool M. R. et al. (2009a). Eeyarestatin I inhibits Sec61-mediated protein translocation at the endoplasmic reticulum. *J. Cell. Sci.* 122, 4393-4400. 10.1242/jcs.054494 19903691 PMC2779136

[JCS198655C9] Cross B. C. S., Sinning I., Luirink J. and High S. (2009b). Delivering proteins for export from the cytosol. *Nat. Rev. Mol. Cell Biol.* 10, 255-264. 10.1038/nrm2657 19305415

[JCS198655C10] Devaraneni P. K., Conti B., Matsumura Y., Yang Z., Johnson A. E. and Skach W. R. (2011). Stepwise insertion and inversion of a type II signal anchor sequence in the ribosome-Sec61 translocon complex. *Cell* 146, 134-147. 10.1016/j.cell.2011.06.004 21729785 PMC3181170

[JCS198655C11] Elliott J. G., Oliver J. D. and High S. (1997). The thiol-dependent reductase ERp57 interacts specifically with N-glycosylated integral membrane proteins. *J. Biol. Chem.* 272, 13849-13855. 10.1074/jbc.272.21.13849 9153243

[JCS198655C12] Garrison J. L., Kunkel E. J., Hegde R. S. and Taunton J. (2005). A substrate-specific inhibitor of protein translocation into the endoplasmic reticulum. *Nature* 436, 285-289. 10.1038/nature03821 16015336

[JCS198655C13] George K. M., Chatterjee D., Gunawardana G., Welty D., Hayman J., Lee R. and Small P. L. (1999). Mycolactone: a polyketide toxin from Mycobacterium ulcerans required for virulence. *Science* 283, 854-857. 10.1126/science.283.5403.854 9933171

[JCS198655C14] Gilmore R., Blobel G. and Walter P. (1982a). Protein translocation across the endoplasmic reticulum. I. Detection in the microsomal membrane of a receptor for the signal recognition particle. *J. Cell Biol.* 95, 463-469. 10.1083/jcb.95.2.463 6292235 PMC2112970

[JCS198655C15] Gilmore R., Walter P. and Blobel G. (1982b). Protein translocation across the endoplasmic reticulum. II. Isolation and characterization of the signal recognition particle receptor. *J. Cell Biol.* 95, 470-477. 10.1083/jcb.95.2.470 6292236 PMC2112977

[JCS198655C16] Gilmore R., Collins P., Johnson J., Kellaris K. and Rapiejko P. (1991). Transcription of full-length and truncated mRNA transcripts to study protein translocation across the endoplasmic reticulum. *Methods Cell Biol.* 34, 223-239. 10.1016/S0091-679X(08)61683-0 1943802

[JCS198655C17] Goder V. and Spiess M. (2001). Topogenesis of membrane proteins: determinants and dynamics. *FEBS Lett.* 504, 87-93. 10.1016/S0014-5793(01)02712-0 11532438

[JCS198655C18] Gogala M., Becker T., Beatrix B., Armache J.-P., Barrio-Garcia C., Berninghausen O. and Beckmann R. (2014). Structures of the Sec61 complex engaged in nascent peptide translocation or membrane insertion. *Nature* 506, 107-110. 10.1038/nature12950 24499919

[JCS198655C19] Görlich D., Prehn S., Hartmann E., Kalies K.-U. and Rapoport T. A. (1992). A mammalian homolog of SEC61p and SECYp is associated with ribosomes and nascent polypeptides during translocation. *Cell* 71, 489-503. 10.1016/0092-8674(92)90517-G 1423609

[JCS198655C20] Hall B. and Simmonds R. (2014). Pleiotropic molecular effects of the Mycobacterium ulcerans virulence factor mycolactone underlying the cell death and immunosuppression seen in Buruli ulcer. *Biochem. Soc. Trans.* 42, 177-183. 10.1042/BST20130133 24450648

[JCS198655C21] Hall B. S., Hill K., McKenna M., Ogbechi J., High S., Willis A. E. and Simmonds R. E. (2014). The pathogenic mechanism of the Mycobacterium ulcerans virulence factor, mycolactone, depends on blockade of protein translocation into the ER. *PLoS Pathog.* 10, e1004061 10.1371/journal.ppat.1004061 24699819 PMC3974873

[JCS198655C22] Harant H., Lettner N., Hofer L., Oberhauser B., de Vries J. E. and Lindley I. J. D. (2006). The translocation inhibitor CAM741 interferes with vascular cell adhesion molecule 1 signal peptide insertion at the translocon. *J. Biol. Chem.* 281, 30492-30502. 10.1074/jbc.M607243200 16914554

[JCS198655C23] Hentzen D., Mandel P. and Garel J.-P. (1972). Relation between aminoacyl-tRNA stability and the fixed amino acid. *Biochim. Biophys. Acta* 281, 228-232. 10.1016/0005-2787(72)90174-8 4629424

[JCS198655C24] Hessa T., Meindl-Beinker N. M., Bernsel A., Kim H., Sato Y., Lerch-Bader M., Nilsson I., White S. H. and von Heijne G. (2007). Molecular code for transmembrane-helix recognition by the Sec61 translocon. *Nature* 450, 1026-1030. 10.1038/nature06387 18075582

[JCS198655C25] High S. and Dobberstein B. (1992). Mechanisms that determine the transmembrane disposition of proteins. *Curr. Opin. Cell Biol.* 4, 581-586. 10.1016/0955-0674(92)90075-N 1419038

[JCS198655C26] High S. and Tanner M. J. A. (1987). Human erythrocyte membrane sialoglycoprotein beta. The cDNA sequence suggests the absence of a cleaved N-terminal signal sequence. *Biochem. J.* 243, 277-280. 10.1042/bj2430277 3606576 PMC1147844

[JCS198655C27] High S., Andersen S. S., Görlich D., Hartmann E., Prehn S., Rapoport T. A. and Dobberstein B. (1993). Sec61p is adjacent to nascent type I and type II signal-anchor proteins during their membrane insertion. *J. Cell Biol.* 121, 743-750. 10.1083/jcb.121.4.743 8491769 PMC2119797

[JCS198655C28] Johnson N., Vilardi F., Lang S., Leznicki P., Zimmermann R. and High S. (2012). TRC40 can deliver short secretory proteins to the Sec61 translocon. *J. Cell. Sci.* 125, 3612-3620. 10.1242/jcs.102608 22505607 PMC3445324

[JCS198655C29] Johnson N., Hassdenteufel S., Theis M., Paton A. W., Paton J. C., Zimmermann R. and High S. (2013). The signal sequence influences post-translational ER translocation at distinct stages. *PLoS ONE* 8, e75394 10.1371/journal.pone.0075394 24130708 PMC3793985

[JCS198655C30] Junne T., Wong J., Studer C., Aust T., Bauer B. W., Beibel M., Bhullar B., Bruccoleri R., Eichenberger J., Estoppey D. et al. (2015). Decatransin, a new natural product inhibiting protein translocation at the Sec61/SecYEG translocon. *J. Cell. Sci.* 128, 1217-1229. 10.1242/jcs.165746 25616894 PMC4359925

[JCS198655C31] Kalies K.-U. and Römisch K. (2015). Inhibitors of protein translocation across the ER membrane. *Traffic* 16, 1027-1038. 10.1111/tra.12308 26122014

[JCS198655C32] Kida Y., Sakaguchi M., Fukuda M., Mikoshiba K. and Mihara K. (2000). Membrane topogenesis of a type I signal-anchor protein, mouse synaptotagmin II, on the endoplasmic reticulum. *J. Cell Biol.* 150, 719-730. 10.1083/jcb.150.4.719 10952998 PMC2175286

[JCS198655C34] Kocik L., Junne T. and Spiess M. (2012). Orientation of internal signal-anchor sequences at the Sec61 translocon. *J. Mol. Biol.* 424, 368-378. 10.1016/j.jmb.2012.10.010 23084973

[JCS198655C35] Kutay U., Ahnert-Hilger G., Hartmann E., Wiedenmann B. and Rapoport T. A. (1995). Transport route for synaptobrevin via a novel pathway of insertion into the endoplasmic reticulum membrane. *EMBO J.* 14, 217-223. 7835332 10.1002/j.1460-2075.1995.tb06994.x PMC398073

[JCS198655C36] MacKinnon A. L., Paavilainen V. O., Sharma A., Hegde R. S. and Taunton J. (2014). An allosteric Sec61 inhibitor traps nascent transmembrane helices at the lateral gate. *Elife* 3, e01483 10.7554/eLife.01483 24497544 PMC3913039

[JCS198655C37] Maifeld S. V., MacKinnon A. L., Garrison J. L., Sharma A., Kunkel E. J., Hegde R. S. and Taunton J. (2011). Secretory protein profiling reveals TNF-α inactivation by selective and promiscuous Sec61 modulators. *Chem. Biol.* 18, 1082-1088. 10.1016/j.chembiol.2011.06.015 21944747 PMC3855466

[JCS198655C38] Martoglio B., Hofmann M. W., Brunner J. and Dobberstein B. (1995). The protein-conducting channel in the membrane of the endoplasmic reticulum is open laterally toward the lipid bilayer. *Cell* 81, 207-214. 10.1016/0092-8674(95)90330-5 7736572

[JCS198655C39] McKenna M., Simmonds R. E. and High S. (2016). Mechanistic insights into the inhibition of Sec61-dependent co- and post-translational translocation by mycolactone. *J. Cell. Sci.* 129, 1404-1415. 10.1242/jcs.182352 26869228 PMC4852723

[JCS198655C40] Meindl-Beinker N. M., Lundin C., Nilsson I. M., White S. H. and von Heijne G. (2006). Asn- and Asp-mediated interactions between transmembrane helices during translocon-mediated membrane protein assembly. *EMBO Rep.* 7, 1111-1116. 10.1038/sj.embor.7400818 17008929 PMC1679787

[JCS198655C41] Mothes W., Prehn S. and Rapoport T. A. (1994). Systematic probing of the environment of a translocating secretory protein during translocation through the ER membrane. *EMBO J.* 13, 3973-3982. 8076593 10.1002/j.1460-2075.1994.tb06713.x PMC395317

[JCS198655C42] Ogbechi J., Ruf M.-T., Hall B. S., Bodman-Smith K., Vogel M., Wu H.-L., Stainer A., Esmon C. T., Ahnström J., Pluschke G. et al. (2015). Mycolactone-dependent depletion of endothelial cell thrombomodulin is strongly associated with fibrin deposition in buruli ulcer lesions. *PLoS Pathog.* 11, e1005011 10.1371/journal.ppat.1005011 26181660 PMC4504485

[JCS198655C43] Oliver J., Jungnickel B., Görlich D., Rapoport T. and High S. (1995). The Sec61 complex is essential for the insertion of proteins into the membrane of the endoplasmic reticulum. *FEBS Lett.* 362, 126-130. 10.1016/0014-5793(95)00223-V 7720858

[JCS198655C44] Paatero A. O., Kellosalo J., Dunyak B. M., Almaliti J., Gestwicki J. E., Gerwick W. H., Taunton J. and Paavilainen V. O. (2016). Apratoxin kills cells by direct blockade of the sec61 protein translocation channel. *Cell Chem. Biol.* 23, 561-566. 10.1016/j.chembiol.2016.04.008 27203376

[JCS198655C45] Pahlevan A. A., Wright D. J., Andrews C., George K. M., Small P. L. and Foxwell B. M. (1999). The inhibitory action of Mycobacterium ulcerans soluble factor on monocyte/T cell cytokine production and NF-kappa B function. *J. Immunol.* 163, 3928-3935. 10490994

[JCS198655C46] Perin M. S., Brose N., Jahn R. and Südhof T. C. (1991). Domain structure of synaptotagmin (p65). *J. Biol. Chem.* 266, 623-629. 1985919

[JCS198655C47] Pfeffer S., Burbaum L., Unverdorben P., Pech M., Chen Y., Zimmermann R., Beckmann R. and Förster F. (2015). Structure of the native Sec61 protein-conducting channel. *Nat. Commun.* 6, 8403 10.1038/ncomms9403 26411746 PMC4598622

[JCS198655C48] Pitonzo D., Yang Z., Matsumura Y., Johnson A. E. and Skach W. R. (2009). Sequence-specific retention and regulated integration of a nascent membrane protein by the endoplasmic reticulum Sec61 translocon. *Mol. Biol. Cell* 20, 685-698. 10.1091/mbc.E08-09-0902 19019984 PMC2626564

[JCS198655C49] Shaw A. S., Rottier P. J. and Rose J. K. (1988). Evidence for the loop model of signal-sequence insertion into the endoplasmic reticulum. *Proc. Natl. Acad. Sci. USA* 85, 7592-7596. 10.1073/pnas.85.20.7592 2845415 PMC282238

[JCS198655C50] Silva M. T., Portaels F. and Pedrosa J. (2009). Pathogenetic mechanisms of the intracellular parasite Mycobacterium ulcerans leading to Buruli ulcer. *Lancet Infect. Dis.* 9, 699-710. 10.1016/S1473-3099(09)70234-8 19850228

[JCS198655C51] Simmonds R. E., Lali F. V., Smallie T., Small P. L. C. and Foxwell B. M. (2009). Mycolactone inhibits monocyte cytokine production by a posttranscriptional mechanism. *J. Immunol.* 182, 2194-2202. 10.4049/jimmunol.0802294 19201873

[JCS198655C52] Song F., Fidanze S., Benowitz A. B. and Kishi Y. (2002). Total synthesis of the mycolactones. *Org. Lett.* 4, 647-650. 10.1021/ol0172828 11843613

[JCS198655C53] Torrado E., Adusumilli S., Fraga A. G., Small P. L. C., Castro A. G. and Pedrosa J. (2007). Mycolactone-mediated inhibition of tumor necrosis factor production by macrophages infected with Mycobacterium ulcerans has implications for the control of infection. *Infect. Immun.* 75, 3979-3988. 10.1128/IAI.00290-07 17517872 PMC1951989

[JCS198655C54] Trueman S. F., Mandon E. C. and Gilmore R. (2012). A gating motif in the translocation channel sets the hydrophobicity threshold for signal sequence function. *J. Cell Biol.* 199, 907-918. 10.1083/jcb.201207163 23229898 PMC3518225

[JCS198655C55] Van den Berg B., Clemons W. M., Collinson I., Modis Y., Hartmann E., Harrison S. C. and Rapoport T. A. (2004). X-ray structure of a protein-conducting channel. *Nature* 427, 36-44. 10.1038/nature02218 14661030

[JCS198655C57] von Heijne G. (1985). Signal sequences. The limits of variation. *J. Mol. Biol.* 184, 99-105. 10.1016/0022-2836(85)90046-4 4032478

[JCS198655C58] von Heijne G. (1986). Towards a comparative anatomy of N-terminal topogenic protein sequences. *J. Mol. Biol.* 189, 239-242. 10.1016/0022-2836(86)90394-3 3783674

[JCS198655C59] Voorhees R. M. and Hegde R. S. (2016). Structure of the Sec61 channel opened by a signal sequence. *Science* 351, 88-91. 10.1126/science.aad4992 26721998 PMC4700591

[JCS198655C60] Wahlberg J. M. and Spiess M. (1997). Multiple determinants direct the orientation of signal-anchor proteins: the topogenic role of the hydrophobic signal domain. *J. Cell Biol.* 137, 555-562. 10.1083/jcb.137.3.555 9151664 PMC2139883

[JCS198655C61] Walsh D. S., Portaels F. and Meyers W. M. (2011). Buruli ulcer: advances in understanding Mycobacterium ulcerans infection. *Dermatol. Clin.* 29, 1-8. 10.1016/j.det.2010.09.006 21095521

[JCS198655C62] Walter P. and Lingappa V. R. (1986). Mechanism of protein translocation across the endoplasmic reticulum membrane. *Annu. Rev. Cell Biol.* 2, 499-516. 10.1146/annurev.cb.02.110186.002435 3030381

[JCS198655C63] Walter P., Ibrahimi I. and Blobel G. (1981). Translocation of proteins across the endoplasmic reticulum. I. Signal recognition protein (SRP) binds to *in-vitro*-assembled polysomes synthesizing secretory protein. *J. Cell Biol.* 91, 545-550. 10.1083/jcb.91.2.545 7309795 PMC2111968

